# Surface Plasmon Resonance and Bending Loss-Based U-Shaped Plastic Optical Fiber Biosensors

**DOI:** 10.3390/s18020648

**Published:** 2018-02-22

**Authors:** Ariadny da S. Arcas, Fábio da S. Dutra, Regina C. S. B. Allil, Marcelo M. Werneck

**Affiliations:** 1 Nanotechnology Engineering Program, Universidade Federal do Rio de Janeiro, Rio de Janeiro 21941-901, Brazil; ariadny@lif.coppe.ufrj.br (A.d.S.A.); fabiodutra@petrobras.com.br (F.d.S.D.); 2 Petrobras’ Research and Development Center, CENPES, Rio de Janeiro 21941-915, Brazil; 3 Electrical Engineering Program, COPPE, Universidade Federal do Rio de Janeiro, Rio de Janeiro 21941-901, Brazil; regina@lif.coppe.ufrj.br

**Keywords:** biosensor, bacteria detection, bending loss, U-shaped fiber sensor, *Escherichia coli*, plastic optical fiber, refractive index, surface plasmon resonance

## Abstract

*Escherichia coli* (*E. coli*) is a large and diverse bacteria group that inhabits the intestinal tract of many mammals. Most *E. coli* strains are harmless, however some of them are pathogenic, meaning they can make one sick if ingested. By being in the feces of animals and humans, its presence in water and food is used as indicator of fecal contamination. The main method for this microorganism detection is the bacterial culture medium that is time-consuming and requires a laboratory with specialized personnel. Other sophisticated methods are still not fast enough because they require sending samples to a laboratory and with a high cost of analysis. In this paper, a gold-coated U-shaped plastic optical fiber (POF) biosensor for *E. coli* bacteria detection is presented. The biosensor works by intensity modulation principle excited by monochromatic light where the power absorption is imposed by predominant effect of either bending loss or surface plasmon resonance (SPR), depending on the gold thickness. Bacterial selectivity is obtained by antibody immobilization on the fiber surface. The biosensor showed a detection limit of 1.5 × 10^3^ colony-forming units (CFU)/mL, demonstrating that the technology can be a portable, fast response and low-cost alternative to conventional methodologies for quality analysis of water and food.

## 1. Introduction

Annually, thousands of people are hospitalized for foodborne illnesses, and some so severe that they result in death [1]. Foodborne diseases have increased significantly worldwide [2] having been motivated by several factors such as poor sanitation and food hygiene [3], population growth and disorderly urbanization, manufacturing contamination in industry due to bad practices, and the need for large-scale food production [4,5].

Among the microorganisms that cause foodborne diseases, the most common is the *Escherichia coli* (also known as *E. coli*) bacterium. *E. coli* is a large and diverse bacteria group that usually inhabits the intestinal tract of humans and other warm-blooded mammals. Most *E. coli* strains are harmless, however others are pathogenic, meaning that they can produce some kind of sickness if ingested [6]. By being in the feces of animals and humans, its presence in water and food is used as an indicator of fecal contamination.

The most common routes of *E. coli* contamination are drinking water, vegetables, undercooked meat, unpasteurized milk, and by bathing in polluted rivers and seas [7,8]. Its importance as a public health problem is evident in *E. coli* outbreaks such as those that occurred in July 1996 in Osaka schools, Japan [9,10,11], in 1982 in the Oregon and Michigan cities, United States [12], and in May 2000 in the Ontario Walkerton farming community, Canada [13].

The main method for this microorganism detection is bacterial culture medium, which is time-consuming and requires a laboratory with specialized personnel. Other sophisticated methods are still not fast enough because they require sending samples to a laboratory, and involve high cost of analysis [14,15,16,17].

In this sense, great efforts are being made in the last two decades to develop new portable, fast-response, and reliable biosensor technologies for substituting conventional detection methods. Among these, optical technology has emerged on the market as a powerful, cheap and reliable tool allowing for the development of new techniques for biological detection.

Optical biosensors offer several advantages when compared with their conventional electric counterparts such as electric passiveness, long distance sensing, the possibility of multiplexing several sensing channels, electromagnetic immunity, etc. Baccar et al. [18], for instance, developed a simple low cost nanobiosensor by surface plasmon resonance (SPR) with a gold thin film deposited on a prism for *E. coli* detection with a detection limit of 10^3^ colony-forming units CFU/mL. Geng et al. [16], as example, developed a fiber optic biosensor by evanescent wave absorption for *E. coli* O157:H7 detection in minced meat. The biosensor was able to measure 10^4^ CFU/mL of bacteria concentration after four hours. Wandermur et al. [19] created a plastic optical fiber (POF) biosensor to detect *E. coli* O55 by evanescent wave (EW) principle. The sensor’s detection limit was 10^4^ CFU/mL after 60 min. Halkare et al. [20] produced a localized surface plasmon resonance (LSPR) based on a U-shaped fiber-optic probe for detection of *E. coli* B40 using bacteriophage T4. The sensor was able to detect a bacteria concentration of 10^8^ CFU/mL. 

This work presents a POF sensor coated with gold thin film for *E. coli* detection as a portable, fast response and low-cost alternative to conventional detection methodologies. The biosensor was manufactured in a U-shaped format and works by intensity-modulation principle excited by a monochromatic light that is attenuated by bending loss and SPR.

The U-shaped POF sensor detects the changes in the refractive index (RI) of the surrounding medium. The sensor is functionalized with *E. coli* antibodies attached to the gold thin film. When bacteria are present in the sample, they become attached to the sensor’s surface, increasing the RI and changing the SPR resonance frequency.

The biosensor was experimentally tested under several bacteria concentrations and presented a detection limit of 1.5 × 10^3^ CFU/mL, demonstrating that the technology might be an efficient tool for quality analysis of water and food.

## 2. Working Principle 

A bacterium presents a RI about 1.39 at 400–800 nm [15,21,22], slightly higher than that of pure water (1.333). Nonetheless, even in heavily contaminated water, with 10^8^ CFU/mL, for instance, the bacteria concentration is not high enough to substantially change the RI of the water. Therefore, in order to detect bacteria by RI sensing, the detection system needs to concentrate the target bacteria around the fiber.

The technique known as immunocapture consists in covalently bonding the specific antibody on the sensor sensitive region in such a way that only bacteria of specific species are captured and fixed on the sensor surface [19,23,24]. 

The proposed sensor works by the measurement principle of optical power loss in a U-shaped POF. The light conducted by the optical fiber is attenuated, depending on the refractive index of the medium surrounding the sensor. In the shaped region, two effects cause light power losses: bending loss and SPR. SPR is the resonant oscillation of conduction electrons at the interface between a noble metal and a dielectric material, stimulated by incident light [25,26]. The SPR effect depends on the light wavelength, the incident angle, film thickness, and the surrounding RI [27,28,29].

In a multimode optical fiber, the light propagation takes place by the principle of total internal reflection (TIR); as a consequence, the incidence angle of the traveling optical ray is required to be higher than that of the critical angle. In the core-cladding interface, an EW is generated that extends with a range from 50–1000 nm into the cladding depending on the wavelength, the refractive index, and the angle of incidence [30].

**Figure 1:**
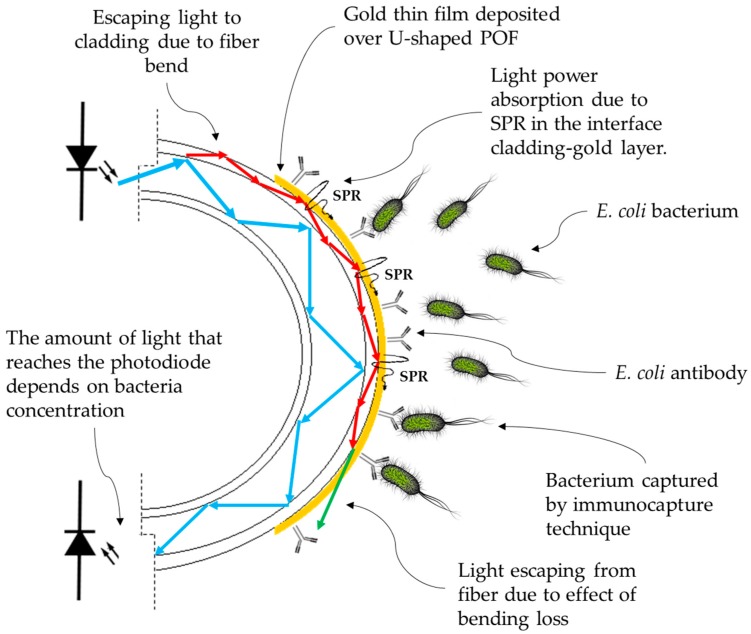
Illustration of the U-shaped gold-coated biosensor working principle.

At a fiber bend, the higher order propagation modes (the guided light rays reflecting at angles close to the critical angle) reach the core-cladding interface at an angle smaller than the critical one, and refract into the cladding instead of reflecting by TIR. At the cladding interface with the surrounding medium, there is another critical angle, so that all cladding modes that reach this interface at smaller angles are lost into the surrounding medium. Thus, an increase in RI in the surrounding of a U-shaped POF increases the critical angle of the cladding interface, which results in decreasing the V number (maximum number of modes allowed inside the fiber) and, as a consequence, more attenuation of the light. The attenuation phenomenon by the fiber bend is known as bending loss. An illustration of the bare U-shaped POF probe operating by bending loss effect in the presence of bacteria is showed in Figure 1 [23,31,32,33,34].

By covering the U-shaped POF probes with gold film, if the layer is thin enough to be transparent to some wavelengths, the attenuation is still predominantly imposed by the effect of bending loss. In this case, the sensor can be used in the same way as a pristine fiber, but it is endowed with a layer of gold for better immobilization of antibodies. However, for specific wavelengths, the SPR effect absorbs these wavelengths, causing light attenuation. 

An illustration of a gold-coated U-shaped POF probe operating by the SPR effect in the presence of bacteria is shown in Figure 1.

## 3. Materials and Methods

### 3.1. Probe Fabrication

**Figure 2:**
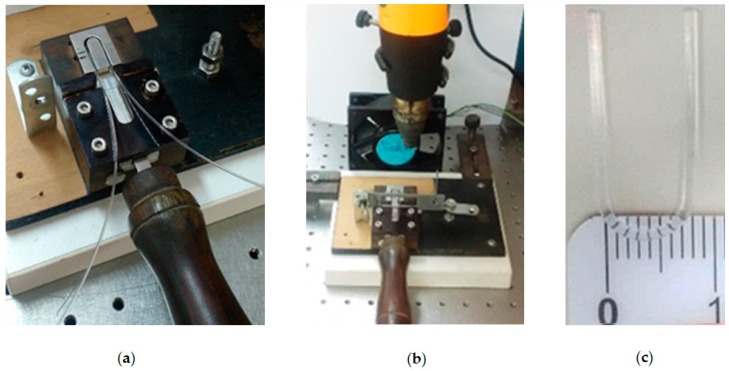
(**a**) Custom-made device used for U-shaped plastic optical fiber (POF) probe molding; (**b**) Hot-air blower gun used for heating; (**c**) The finished U-shaped POF fiber with 8 mm in diameter.

U-shaped probes were fabricated using Mitsubishi Rayon Eska GH 4001 multi-mode POF 1 mm in diameter and a cladding thickness of 10 μm. The core material was polymethyl methacrylate (PMMA) with RI = 1.49 in the visible range and the cladding material was fluorinated polymer with RI = 1.41. The fabrication process consisted of cutting the POF into approximately 10 cm-long sections, and both end surfaces were cleaved and polished for maximum light coupling. Then, the 10 cm-long sections were shaped and placed in a manually operated custom-made device (Figure 2a) for molding the U-shaped POF in a curve with 8 mm in diameter (Figure 2c). The fiber was heated by a hot-air blower gun (Figure 2b) for 15 s. During the molding procedure, the temperature was maintained below 70 °C so that the fiber melting point would not be reached.

**Figure 3:**
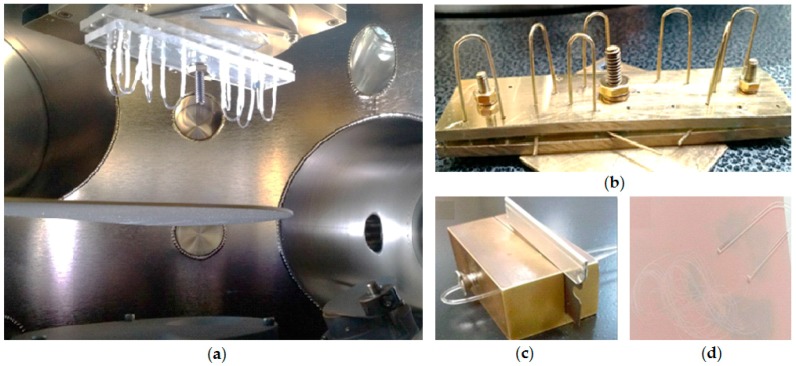
(**a**) Bare U-shaped POF probes installed inside the vacuum sputtering chamber 10 cm above gold target and tilted 45° from vertical; (**b**) U-shaped POF probes coated with 70 nm gold thin film; (**c**) Custom-made device used to cleave the probe ends; (**d**) End faces were polished with a 3 µm polish film in “figure of eight” movements.

A radio-frequency (RF) magnetron sputtering system (Aja International, Scituate, MA, USA) was used for gold thin film deposition on the U-shaped probe surface (Figure 3). A 40 W RF power was applied to ionize the argon gas (plasma) inside the vacuum sputtering chamber. The argon flow rate was maintained at 12 sccm (standard cubic centimeters per minute) at a pressure of 4 × 10^−3^ mbar. The gold target size was 25.4 mm in diameter and 0.3 mm in thickness. 

After molding, the U-shaped probes were cleaned with isopropyl alcohol for two minutes (not exceeding this time in order to avoid fiber cracking), washed in ultrapure water, and dried with ultrapure nitrogen. Then, they were attached to an acrylic support fixed in the substrate holder 10 cm above the gold target and tilted 45° from vertical, as showed in Figure 3a. The substrate holder was rotated at 20 rpm during the deposition process to improve film thickness uniformity. Under these conditions, the gold deposition rate is about 3.5 nm/min and layers of 3.5, 7.0, 10, 18, 30, 50, 70, and 100 nm were deposited in several U-shaped probes (Figure 3b). Notice that due to the fiber cylindrical geometry, the film deposition was not uniform. 

After deposition, probes ends were cleaved with the device showed in Figure 3c and polished with a 3-µm polish film as illustrated in Figure 3d.

### 3.2. Optoelectronic Setups

Two optoelectronic setups were developed. Setup 1 consisted of an 880 nm LED (SFH485P, Siemens) as light source driven by a current source controlled by an Arduino Uno microcontroller. The reason for using an IR light source was to match with the highest sensitivity of the Si photodetector. The light transmitted by the U-shaped POF probe was received by a photodiode, also processed by the microcontroller. Both the LED and the photodiode were encapsulated in an opaque casing to avoid external interference and light leakage of the system.

**Figure 4:**
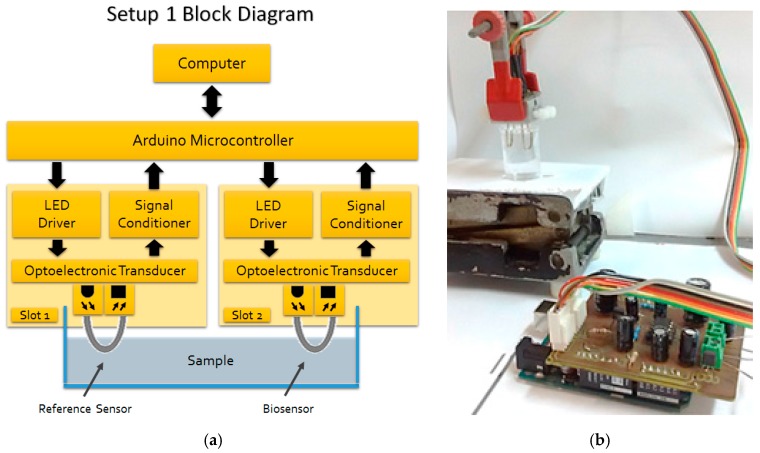
(**a**) Setup 1 block diagram. There are two slots, one for the reference sensor and the other one for the bacteria sensing sensor; (**b**) Setup 1 picture.

The Arduino communicates via USB port with a microcomputer, in which there is a digital processing software. The Setup 1 allows simultaneous reading of two sensors in order to allow one of them to be used as a reference sensor without antibody immobilization, and the other one as a bacteria sensor for comparative effect. Setup 1 block diagram and its picture are shown in Figure 4a,b, respectively.

Setup 2 consisted of a 350–845 nm spectrometer and a white light source (HR-4000, HL-2000, Ocean Optics, Winter Park, FL, USA) connected at each end of the U-shaped probe. The reason for adopting Setup 2 (with white light source and a monochromator) was to check for the best wavelength, as Setup 1 used an 880 nm LED.

**Figure 5:**
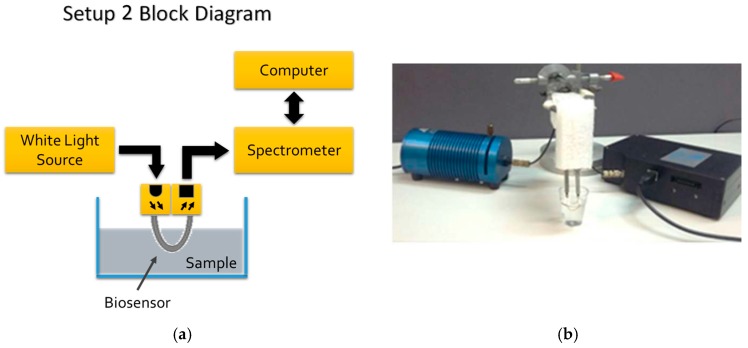
(**a**) Setup 2 block diagram; (**b**) Setup 2 picture. The white light source is at the left and the spectrometer at the right.

The Setup 2 block diagram and its picture are shown in Figure 5a,b, respectively.

### 3.3. Sucrose Solutions for RI Testing

Aqueous sucrose solutions with RI varying from 1.33–1.39 were prepared in ultrapure water for evaluating sensors sensitivity and repeatability. The solutions refractive index was measured at 589 nm wavelength by an Abbe refractometer (Q767BD, Quimis, Diadema, SP, Brazil) with an uncertainty of 0.0002 refractive index unit (RIU).

The gold-coated U-shaped probes with gold thickness of 18 nm or less were tested with the Setup 1 in eight sucrose solutions with RI varying from 1.33–1.39, randomly. For each concentration, 20 readouts were taken. In turn, the gold-coated U-shaped probes with gold thickness of 30 nm or more were tested in five sucrose solutions, also randomly. Twenty readouts were carried out for each concentration.

With Setup 2, gold-coated U-shaped probes with 10 nm and 70 nm gold thicknesses were tested in six sucrose solutions with RI varying from 1.33–1.39, randomly. For each concentration, 30 readouts were taken.

Straight lines were fitted to experimental data by linear regression for determining a best linear relationship for sensor response versus RI. The expanded uncertainties with 95% confidence level were obtained by the mean standard deviations multiplied by the Student’s t-distribution k-factors, referring to the degrees of freedom used in the tests.

### 3.4. Antibody Immobilization Protocol

The U-shaped POF probes were immobilized with rabbit anti-*Escherichia coli* polyclonal antibody (Bio-Rad, Hercules, CA, USA). The antibody immobilization protocol was adapted from [35], which is briefly described here.

First, the probes were cleaned in isopropyl alcohol P.A. for 2 min and after that in ultrapure water. Then, the bends of the probes were immersed in a 4 mM cysteamine solution for 1.5 h. In this step, thiol (-SH) functional groups of cysteamine molecule bind exchangeable with gold surface through strong ion-exchange interactions. Thereafter, the probes were washed first with absolute ethyl alcohol and then in ultrapure water twice.

The next step was the immobilization of the antibodies by binding the cysteamine amine (-NH_2_) functional groups to the carboxylic acid (-COOH) functional groups present in the *E. coli* antibodies. In order to do so, a solution of 100 mM 1-Ethyl-3-(3-dimethylaminopropyl) carbodiimide (EDAC) and 40 mM *N*-Hydroxysuccinimide (NHS) was dissolved in 10 mM phosphate-buffered saline (PBS). Soon after, an antibody concentration of 10 µg/mL was added in the solution and left to rest during 15 min for activation of the carboxylic acid groups. Thereafter, the probes functionalized with cysteamine were immersed in this solution and left for 1 h for antibody immobilization.

Then, the probes with antibodies were immersed in a 10 mM bovine serum albumin (BSA) solution and left for 30 min in order to block unreacted cysteamine amine functional groups. The removal of unbound reagents is of great importance to avoid non-specific binding thereby reducing the selectivity of the sensor. Lastly, the probes were washed in a 10 mM PBS solution three times to remove unbound antibody molecules. PBS, EDAC and NHS were supplied by Sigma-Aldrich and BSA was supplied by Merck.

### 3.5. Bacterial Suspensions and Testing

Cultures of *E. coli* O55 bacteria (Osvaldo Cruz Foundation, Fiocruz, Rio de Janeiro, RJ, Brazil) were grown in tryptone soya agar (TSA). For selectivity tests, cultures of *Enterobacter cloacae*, *Salmonella typhimurium* bacteria (Laboratory of Food Microbiology, UFRJ, Rio de Janeiro, RJ, Brazil) and *Bacillus subtilis* (Osvaldo Cruz Foundation, Fiocruz, Rio de Janeiro, RJ, Brazil), were grown in soybean triptych broth (TSB) and TSA.

Bacterial suspensions were prepared using ultrapure water and NaCl P.A. (Synth, Brazil). The concentrations were determined using the McFarland 0.5 turbidity standard by visual comparison which correspond to a *E. coli* concentration of 1.5 × 10^8^ CFU/mL [36,37] and confirmed by absorbance measurements in the spectrophotometer (UV-1800, Pro-Tools, Porto Alegre, RS, Brazil). The McFarland 0.5 scale has an absorbance value between 0.08 and 0.1 for a wavelength of 625 nm and optical path of 1 cm [36,37]. All bacterial suspensions were prepared using a solution of 0.85% sodium chloride.

Bacteria suspensions with concentrations from 10^3^–10^8^ CFU/mL were prepared for testing the biosensors. In the Setup 1, the gold-coated U-shaped probes with 10 nm and 70 nm thicknesses were immersed into a 5 mL beaker with bacteria suspension. A bare U-shaped probe was immersed together to acts as a reference. Readouts were carried out at intervals of 30 s or 1 min for up to 60 min. In Setup 2, just the gold-coated U-shaped probes with 70 nm thickness were tested. Readouts were carried out at intervals of 5 min for up to 60 min.

After the bacterial suspension testing, the sensors were submitted to safranin solution for 5 s for cell coloring. Then, they were washed with ultrapure water and left to dry at room temperature. After drying, the sensors were taken to an optical microscope with a 40× magnification. Images were produced with a 13-megapixel camera. The bacterial coverage area was estimated by the ImageJ software.

## 4. Results and Discussion

### 4.1. Gold Thin Film Transmittance

The transmittances of the gold thin films were measured by illuminating gold thin films deposited on glass slides using a white light source (UV to NIR). A pristine glass slide was used as reference. On the other side of the slide a spectrophotometer (Evolution 300, Thermo Scientific, São Paulo, SP, Brazil) analyzed the transmitted light. The light beam was perpendicular to the slide.

**Figure 6:**
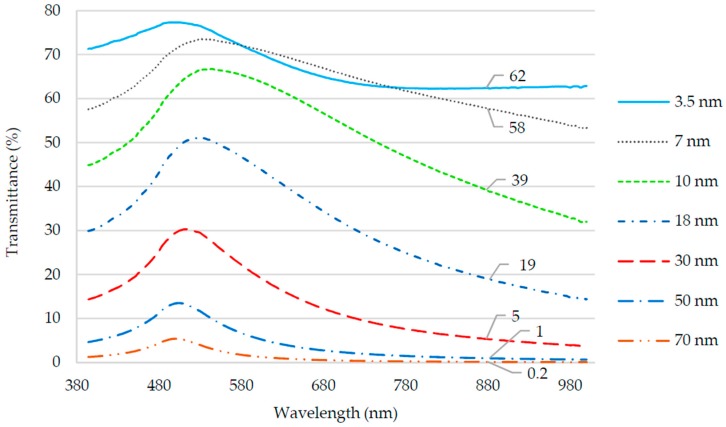
Light spectral transmittance of gold thin films. The transmittance values for 880 nm wavelength (light emitting diode (LED) of Setup 1) for each gold film thickness are shown.

The results are showed in the Figure 6. Note that the light transmittance of 880 nm wavelength LED used by Setup 1 is less than 1% for thicknesses exceeding 50 nm and greater than 19% for thickness less than 18 nm. Therefore, the probes coated with gold layer with thickness greater than 50 nm would not lose light on the bend since the film transmittance was negligible such that practically all the light would be reflected and remain inside the fiber.

Also, note that the transmittance was at maximum at wavelengths close to 500–520 nm for all thicknesses, which is inherent to the gold nature. Thus, the LED wavelength should be in this range if the sensor works by the effect of bending loss. However, an 880 nm LED was used because it was part of Setup 1, which was already available for use. These results were in accordance with [38].

### 4.2. RI Measurements

#### 4.2.1. Sucrose Solutions Testing in Setup 1

**Figure 7:**
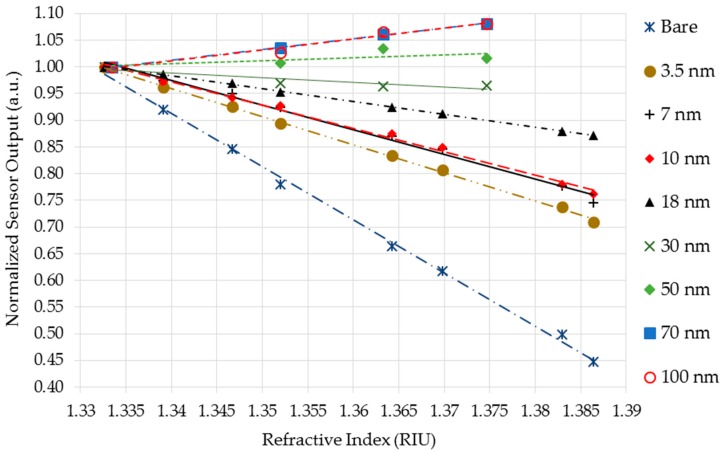
Averaged sensor responses for Setup 1 with reflective index (RI) variations for several gold layer thickness.

Bare and gold-coating U-shaped probes (at least five of each) were tested in sucrose solutions with RI varying from 1.33–1.39 in the Setup 1. The averaged sensor responses are shown in Figure 7. Each dot in the plot is the average of 20 readings. The curve-fitting equations were determined by linear regression. 

It can be clearly seen from plots on the Figure 7 that bare U-shaped probes showed a linear relationship between sensor output voltage and sucrose solutions RI for the whole range, indirectly proportional to RI changes, which agreed with the results of [19,23]. The R^2^ coefficients were greater than 0.99 for all sensors, which demonstrate a good fit of the linear equations with experimental data. The average sensitivity of the sensors was −39.71 V/RIU with a standard deviation of 2.89 (see Table 1).

It is noticed that the gold-coated U-shaped probes also showed linear relationships of output voltage versus RI for thicknesses up to 18 nm. Notice also that the thicker the layer, the smaller the sensitivity. This is due to the higher reflectivity of thicker layers that decreases the EW interaction with the external medium. Table 1 shows a summary of averaged sensitivity and its standard deviation for each thickness, as well as the maximum expanded uncertainty (95% confidence level) of the inverse regression.

It can be seen that the probes coated with 70 nm and 100 nm presented a linear behavior for the range of 1.33–1.38, but as opposed to the response of sensors with thinner layers, the output rose with the RI. In these sensors, the SPR effect predominated over the bending loss effect in such a way that, as SPR absorption decreased as RI increased, the output light increased more than the losses due to fiber bending. These results were in accordance with [39,40,41].

Probes with thicknesses between 30 nm and 50 nm were caught in the middle of the two effects, SPR and bending loss. In this region, the two effects competed with each other so that the output light remained constant over the entire RI range.

In summary, for layers up to 30 nm, the dominant effect was the bending loss in which the output light power decreased with an increase of RI, whereas for layers with thickness of 30 nm and above, the dominant effect was the SPR with an increase of output power as RI increased.

#### 4.2.2. Sucrose Solutions Testing in Setup 2

**Figure 8:**
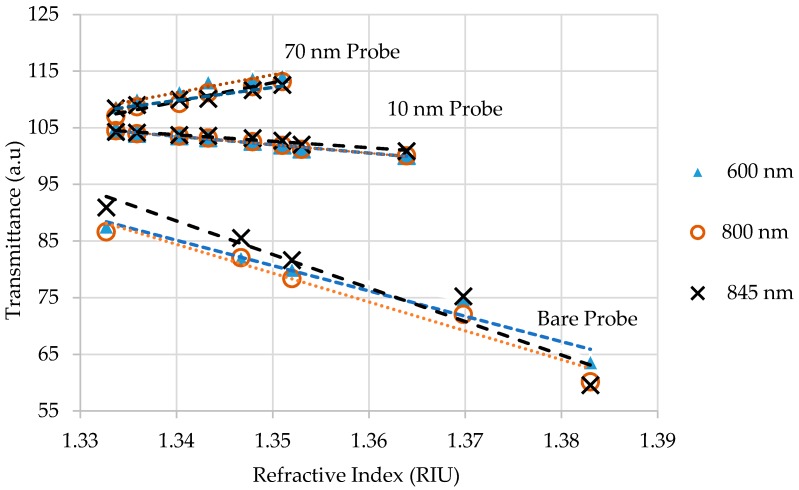
Sensor responses for RI variations using Setup 2 for wavelengths 600 nm, 800 nm and 845 nm for bare probe, 10 nm gold-coated probe and 70 nm gold-coated probe.

Setup 2, as shown in Figure 8, provided sensor responses for RI variations using Setup 2. Tests with aqueous sucrose solutions were carried out with bare, 10 nm, and 70 nm gold-coated probes. The 10 nm gold-coated probe, in which the effect of bending loss was more pronounced, was chosen because it did not compromise the sensitivity, apart from needing a smaller amount of gold. The 70 nm gold-coated probe, in which the SPR effect was more pronounced, was chosen because it had a reasonable linear response for 1.33–1.38 RI range, and used a smaller amount of gold than the one with 100 nm.

Figure 8 shows the sensor responses for 600 nm, 800 nm and 845 nm wavelengths. By observing the graph, it can be noticed that the bare probe presented a linear and indirectly proportional relationship between sensor response and RI changes, as expected. The graph shows that the 10 nm gold-coated sensor responses presented the same behavior as the bare probe sensor for a RI range of 1.33–1.38, however, with a lower sensitivity due to the gold layer absorption. Unlike Setup 1, where the sensitivity of 10 nm gold-coated probe dropped by half with the bare probe, in Setup 2, the sensitivity was four times lower.

Figure 8 also shows the 70 nm gold-coated sensors responses that presented the same behavior in Setup 1, linear and directly proportional at RI variations for the range of 1.33–1.352. The sensitivity was better than that of 10 nm gold-coated probe.

Table 2 shows a summary of the sensitivity and maximum expanded uncertainty (95% of confidence level) of the inverse regression for each gold thickness.

### 4.3. Bacterial Measurements

Probes immobilized with antibodies were tested in different bacterial suspensions in order to evaluate response time and sensitivity for both Setup 1 and Setup 2. For these tests the 10 nm and 70 nm gold-coated U-shaped probes were chosen.

#### 4.3.1. Bacterial Suspensions Testing in Setup 1

**Figure 9:**
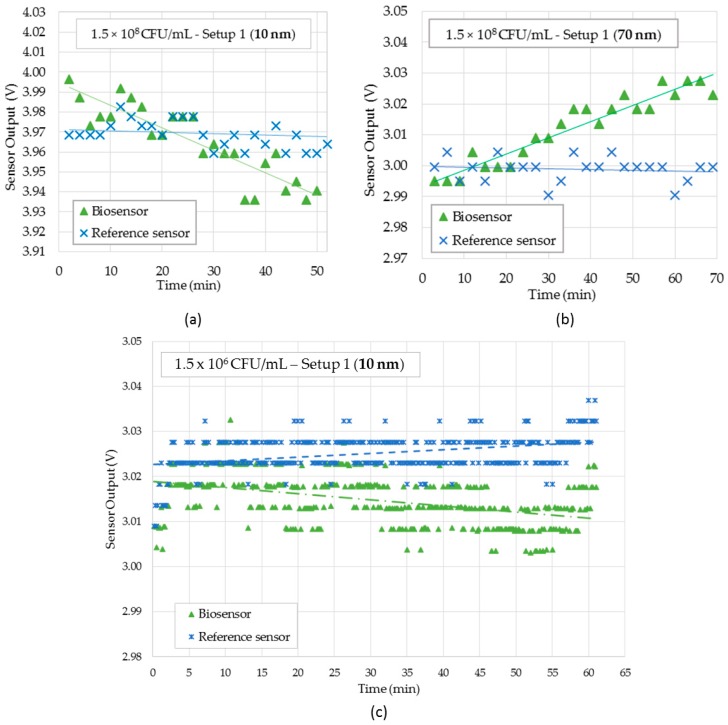
Sensor responses for bacterial suspensions using Setup 1. (**a**) Sensor probe and reference probe for 10 nm gold-coated at 1.5 × 10^8^ CFU/mL bacterial suspension; (**b**) Sensor probe and reference probe for 70 nm gold-coated probes in 1.5 × 10^8^ CFU/mL bacterial suspension; (**c**) Sensor probe and reference for 10 nm gold-coated probes in 1.5 × 10^6^ CFU/mL bacterial suspension.

Figure 9a,b show respectively, the 10 nm and 70 nm gold-coated sensors responses in Setup 1 for a bacteria concentration of 1.5 × 10^8^ CFU/mL. Figure 9c shows the results of 10 nm gold-coated sensor for bacteria concentration of 1.5 × 10^6^ CFU/mL. For lower concentrations, either sensors were unable to detect bacteria with the Setup 1.

Notice in Figure 9a, that the biosensor output voltage decreased over time due to the increase in the surrounding RI caused by immunocapture effect, as the antibody kept capturing the bacteria present in the water. At the same time, the output voltage of reference sensor remained constant, which confirmed the proper operation of the sensor for bacteria detection. As shown in previous works [19,23], U-shaped probes based on bending loss effect had an indirect relationship between sensor response and RI. Nonetheless, in Figure 9b, the sensor output voltage increased in time as more and more bacteria were bound to sensor surface. This behavior was due to the SPR effect at this specific wavelength, previewed by the experiment shown in Figure 9c. These results are in accordance with [26,27].

Figure 9c shows the sensor output of 10 nm gold-coated U-shaped probe for 1.5 × 10^6^ CFU/mL. Notice that the sensor was able to detect this bacterial concentration.

#### 4.3.2. Bacterial Suspensions Testing in Setup 2

**Figure 10:**
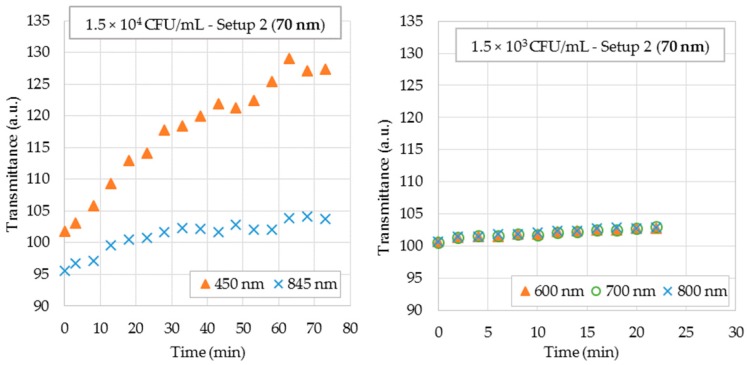
Sensor responses for bacterial suspensions using Setup 2. (**a**) 70 nm gold-coated biosensors in a 1.5 × 10^4^ CFU/mL bacterial suspension; (**b**) 70 nm gold-coated biosensors in a 1.5 × 10^3^ CFU/mL bacterial suspension.

For bacterial suspensions testing in Setup 2, 70 nm gold-coated probes were chosen. Figure 10a,b show the sensor responses for bacteria concentration of 1.5 × 10^4^ CFU/mL and 1.5 × 10^3^ CFU/mL, respectively.

Notice in Figure 10 that the sensor was able to detect a concentration of 1.5 × 10^3^ CFU/mL. The sensor output voltage increased with time due to the increase in the surrounding RI, as expected. As the response was a transmittance spectrum, it was possible to choose the wavelength in which the sensitivity was higher. Tests with 880 nm were not possible as the wavelength range of the spectrometer is limited to 850 nm. It was shown that the system was capable of providing a positive response to the bacterial concentration in less than 10 min. Notice that the higher the bacteria concentration, the more rapidly the output signal rose. 

### 4.4. Selectivity Testing

In order to verify sensor selectivity, tests were carried out with the bacteria *Enterobacter cloacae*, *Salmonella typhimurium* and *Bacillus subtilis*.

**Figure 11:**
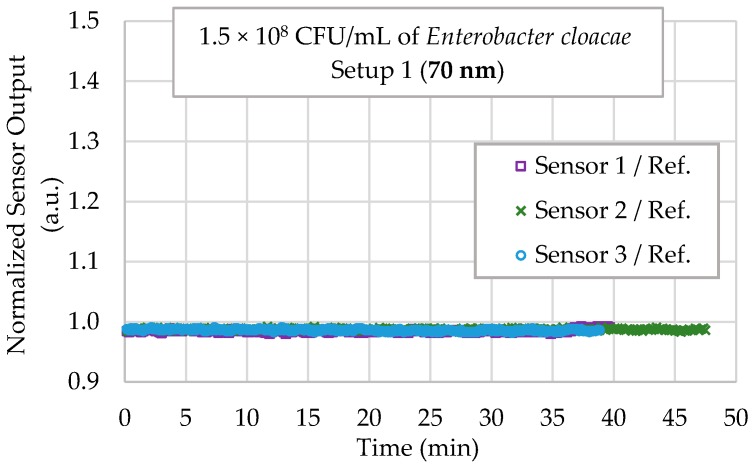
Sensor responses for 70 nm gold-coated U-shaped probe for selectivity testing in a 1.5 × 10^8^ CFU/mL *Enterobacter cloacae* concentration with Setup 1.

In Figure 11, three 70 nm U-shaped biosensors were tested in the Setup 1 with suspensions of the *Enterobacter cloacae* at high concentration (1.5 × 10^8^ CFU/mL). The graphs show results of each sensor. It is observed that, for a time greater than 40 min, the sensors showed no tendency, meaning that the *E. coli* antibody does not capture this species of bacteria, even in high concentration.

**Figure 12:**
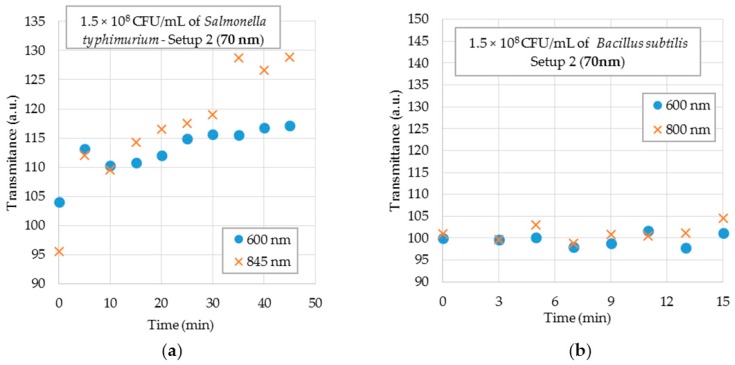
Sensor responses for 70 nm gold-coated probe for selectivity testing in a 1.5 × 10^8^ CFU/mL bacteria concentration with Setup 2 for 600 nm and 845 nm wavelengths. (**a**) *Salmonella typhimurium*; (**b**) *Bacillus subtilis*.

Figure 12a shows the results of the 70 nm probe tested in Setup 2 for *Salmonella typhimurium* suspensions at a concentration of 1.5 × 10^8^ CFU/mL. The graphs show the results for 600 nm and 845 nm wavelengths. It is observed that, in this case, the sensor detected the presence of bacteria other than *E. coli*. It is assumed that this occurred due to the antibody being polyclonal rather than monoclonal, which allowed high specificity and lower cross-reactivity. 

Lastly, Figure 12b shows the results of the 70 nm U-shaped biosensor tested in Setup 2 with *Bacillus subtilis* suspensions at a concentration of 1.5 × 10^8^ CFU/mL. The graphs show the results for 600 nm and 845 nm wavelengths. It is observed that there is no tendency, that is, the sensor was not able to capture the bacteria even in high concentration.

### 4.5. Bacterial Adhesion on Sensor Surface

**Figure 13:**
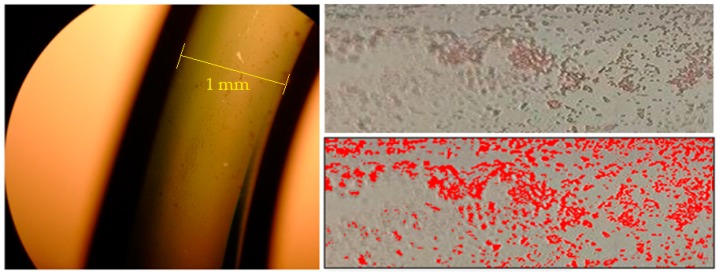
Optical microscope image (40×) of *E. coli* coverage over the 70 nm probe after immersion in a bacteria suspension at 1.5 × 10^8^ CFU/mL concentration. In left, the probe microscope image. In upper right, a 40× magnification image of the bacteria adhered to the fiber surface colored by safranin solution. In bottom right, the coverage area in red estimated by the ImageJ. The coverage area estimated by ImageJ software was 16%.

Figure 13 shows the image obtained by optical microscopy of the bacteria adhesion on the surface of the sensor tested under an *E. coli* concentration of 1.5 × 10^8^ CFU/mL. The coverage area estimated by ImageJ software was 16%. This small coverage indicates that immunocapture could be improved to further reduce the detection limit.

## 5. Conclusions

A gold-coated U-shaped POF biosensor for *E. coli* detection has been presented. This technique allows using POF as a RI sensor even without removing the cladding.

U-shaped probes coated with gold layers up to 18 nm thick present the same behavior as bare U-shaped probes, in which the bending loss is the sensing predominant effect. Probes coated with 30 nm and 50 nm gold layer are not useful for RI sensing in the range of this application. In 70 nm and 100 nm gold-coated probes, the SPR effect is predominant and the biosensor was capable to detect bacteria concentrations as low as of 1.5 × 10^3^ CFU/mL.

In comparing the two employed setups, both detected bacteria. However, Setup 2 was capable to detect up to 10^3^ CFU/mL, whereas Setup 1 the detection limit was 10^6^ CFU/mL.

To the best of our knowledge, there are no other works applying POF sensor in bacteria detection. However, in comparing the results obtained in this work with those from our previous publications [15,19], the sensitivity of bare sensor for Setup 1 was slightly better than those from these mentioned works. Additionally, sensors with gold film in Setup 2 also presented a response for lower bacteria concentration (10^3^ CFU/mL), whereas in [42] the detection limit was 10^8^ CFU/mL and in [19] the detection limit was 10^4^ CFU/mL.

The results demonstrated that the proposed biosensor can be an efficient, low cost, and portable tool for routine analysis of drinkable and bathable water and food quality. This technique can be applied to other types of bacteria by simply immobilizing the antibody of the bacteria to be detected.

In future work, efforts should be devoted to the following aspects: (a) improving the bacteria immobilization and thereby expanding the coverage area on the fiber; (b) a new high-resolution optoelectronic setup operating with an LED with a wavelength that proved to allow greater sensitivity, and (c) selectivity could be improved by using monoclonal instead of polyclonal antibodies.

## Figures and Tables

**Table 1:** Summary of the biosensors average sensitivities, standard deviations and maximum expanded uncertainties (95% confidence level) in Setup 1.

Old Thickness (nm)	Sensitivity (V/RIU)	Standard Deviation (V/RIU)	Uncertainty (RIU)
0	−39.71	2.89	3.5 × 10^−3^
3.5	−20.34	2.25	2.9 × 10^−3^
7	−16.73	2.11	7.4 × 10^−3^
10	−17.57	2.43	9.8 × 10^−3^
18	−9.53	1.06	5.5 × 10^−3^
30	−0.88	--	--
50	0.57	--	--
70	6.01	0.65	1.9 × 10^−2^
100	8.25	1.05	7.7 × 10^−2^

**Table 2:** Summary of the biosensors sensitivities and maximum expanded uncertainties (95% confidence level) in the Setup 2.

Gold Thickness (nm)	Sensitivity (1/RIU)	Uncertainty (RIU)
0	−591.78 (845 nm)	5.8 × 10^−4^
10	−148.66 (600 nm)	5.1 × 10^−4^
70	338.26 (800 nm)	5.6 × 10^−4^
